# Supplementary material for: Multiplexed multicolor antiviral assay amenable for high-throughput research
Source: Nat Commun. 2024 Jan 2;15:42. doi: 10.1038/s41467-023-44339-z (PMC10761739; doi:10.1038/s41467-023-44339-z)
Supplement: Supplementary file 1 — Supplementary Information document - FINAL [file 41467_2023_44339_MOESM1_ESM.pdf]

Multiplexed Multicolor Antiviral Assay Amenable for High-Throughput Research

Li-Hsin Li[1,2], Winston Chiu[1,#], Yun-An Huang[3,4,#], Madina Rasulova[5], Thomas Vercruysse[5,6], Hendrik Jan Thibaut[5], Sebastiaan ter Horst[1,7], Joana Rocha-Pereira[1], Greet Vanhoof[8], Doortje Borrenberghs[9], Olivia Goethals[9], Suzanne J.F. Kaptein[1], Pieter Leyssen[1], Johan Neyts[1] and Kai Dallmeier[1,2] ✉

[1] KU Leuven Department of Microbiology, Immunology and Transplantation, Rega Institute, Laboratory of Virology and Chemotherapy, Leuven, Belgium.

[2] Molecular Vaccinology and Vaccine Discovery group, Leuven, Belgium

[3] KU Leuven Department of Neuroscience, Research Group Neurophysiology, Laboratory for Circuit Neuroscience, Leuven, Belgium.

[4] Vlaams Instituut voor Biotechnologie, Neuro-Electronics Research Flanders (NERF), Leuven, Belgium.

[5] KU Leuven Department of Microbiology, Immunology and Transplantation, Rega Institute, Laboratory of Virology and Chemotherapy, Translational Platform Virology and Chemotherapy (TPVC), Leuven, Belgium.

[6] Current affiliation: AstriVax, Heverlee, Belgium.

[7] Current affiliation: Cerba Research, Rotterdam, The Netherlands.

[8] Janssen Therapeutics Discovery, Janssen Pharmaceutica NV, Beerse, Belgium.

[9] Janssen Global Public Health, Janssen Pharmaceutica NV, Beerse, Belgium.

[#] These authors contributed equally: Winston Chiu and Yun-An Huang

✉ kai.dallmeier@kuleuven.be

Inventory of Supporting Information

Supplementary Methods

Supplementary Tables

Supplementary Table 1:

Optical channel settings for multicolor assay on a 7 color-engine widefield Arrayscan XTI high-content imager (Thermo Fisher Scientific)

Supplementary Figures

Supplementary Fig. 1:

Characteristics of dengue type 2 reporter viruses (DENV-2/X-FPs)

Supplementary Fig. 2:

Growth kinetics of dengue type 2 reporter viruses (DENV-2/X-FPs)

Supplementary Fig. 3:

Growth kinetics of YF17D- and JEV-derived reporter viruses and their parental viruses

Supplementary Fig. 4:

Single virus assay for SNT or antiviral assays

Supplementary Fig. 5:

Fluorescent images of infected V-NIR cells in multi-orthoflavi serum neutralizing assay (SNT) using anti-DENV-2 serum

Supplementary Fig. 6:

Fluorescent images of infected V-NIR cells in multi-orthoflavi serum neutralizing assay (SNT) using anti-JEV serum

Supplementary Fig. 7:

Fluorescent images of infected V-NIR cells in multi-orthoflavi serum neutralizing assay (SNT) using anti-YFV serum

Supplementary Fig. 8:

Correlation analysis and Blant-Altman test (related to Fig. 3f).

Supplementary Fig. 9:

Multiplex flavivirus infections on Huh-7 cells

Supplementary Fig. 10:

Selected inhibitor profiles on the virus RGB model (related to Fig.3)

Supplementary References

Supplementary Methods

Construction of multicolor Dengue type 2 (DENV-2) reporter viruses

The infectious cDNA clones of pShuttle-DV2 and pShuttle-DV2/mCherry have been described[1]. In brief, pShuttle-DV2 was generated by subcloning the genome of DENV-2 New Guinea C strain (NGC strain, GenBank: AF038403.1) from pDVWS501[2] into mammalian expression vector pShuttle-BAC[3]. pShuttle-DV2/mCherry was generated by assembling three cDNA amplicons (A-C) via homologous recombination in yeast, with pShuttle-DV2 serving as template for PCR products *pShuttle-5'UTR-capsid* (A) and *capsid*-prM-E-NS-3'UTR-pShuttle* (B), while pShuttle-YF17D/mCherry[4] serving as template for PCR product *capsid-mCherry-capsid** (C).

Here, a series of pShuttle-DV2 derivatives (pShuttle-DV2/X-FPs) carrying different fluorescent protein genes, namely mAzurite (BFP, blue), eGFP (GFP, green), mCitrine (YFP, yellow), and mMaroon (RFP, dark red) were generated similarly as described[1]. Common fragments A and B were generated by restriction digest of pShuttle-DV2/mCherry to obtain the fragment A (*San*DI, *Mlu*I, and *Xba*I) and fragment B (*Bam*HI). Variable fragment C was generated by PCR (KAPA HiFi HotStart ReadyMix*, Kapa Biosystems*), and cDNA plasmids encoding mAzurite (Addgene, #54617)[5], eGFP (GenBank: HI137399.1), mCitrine (Addgene, #54594)[6], or mMaroon (Addgene, #54554) as templates. Respective amplicons *capsid-Azurite-T2A-capsid**, *capsid-GFP-T2A-capsid**, *capsid-Citrine-T2A-capsid**, and *capsid-Maroon-T2A-capsid** were thus generated by three rounds of amplification using three pairs of primers (pp1-3), with pp1: primer #1 (5'-cgtgtcgactgtacaacagctgacaaagagattctcacttggatccgtgagcaagggcga-3') and primer #2 (5'-ctccacgtcgccacaggtcagcagggacccgcgtccctccttgtacagctcgtccatgcc-3'); pp2: primer #3 (5'-cgagaaatacgcctttcaatatgctgaaacgcgagagaaaccgcgtgtcgactgtacaac-3') and primer #4 (5'-acgggccttctttcgttgattgttaggaccggggttctcctccacgtcgccacaggtcag-3'); and pp3: primer #3 (5'-cgagaaatacgcctttcaatatgctgaaacgcgagagaaaccgcgtgtcgactgtacaac-3') and primer #5 (5'-ccctgttccgttctctcttgagcatgttaaatggagtattacgggccttctttcgttgat-3'). Final pShuttle-DV2/X-FP plasmids were assembled by homologous recombination in *Saccharomyces cerevisiae* yeast (strain YPH500)[7], followed by transformation into competent *E. coli* cells (EPI300-T, Epicenter). Colonies were selected on 20 μg/mL chloramphenicol for amplification as previously described[1]. All constructs were sequence verified by Sanger sequencing.

Recombinant virus production and amplification of virus stocks

As previously described[1], all virus stocks were generated in C6/36 except for the stock of YF17D/mCherry, which was generated in Vero E6. In brief, plasmid of each infectious clone was transfected into BHK-21J cells (Mirus TransIT-LT1). The supernatant was collected at 4 days post transfection (dpt) and added on Vero E6 cells for 5-7 days for virus rescue, depending on the expression of fluorescent signals and virus-induced cytopathic effect (CPE). YF17D/mCherry was harvested at this step to serve as virus stock. All other viruses were overlaid on mosquito cells (C6/36) and incubated for 10–12 days for virus amplification as virus stocks. Virus titers were determined by plaque assay on BHK21J cells as described[4,8], with the incubation period for DENV-2 is 7 days and for both JEV and YFV is 5 days.

Virus titers of PIV1/eGFP, CHIKV/mCherry, and BUNV/mCherry virus stocks were determined on Vero E6 cells as median tissue culture infectious doses ($TCID_{50}$). For the calculations of MOIs in subsequent infection experiments, $TCID_{50}$/mL values obtained were converted operationally into PFU/mL by multiplication using a correction factor of 0.7.

Generation of V-NIR and A549-NIR cells

Vero E6 cells constitutively expressing monomeric near infrared fluorescent protein miRFP-703 (V-NIR) as translational fusion to histone protein H2B were generated by lentiviral transduction. To that end, pLenti-H2B-miRFP703-2A-Blasticidin was modified from the pLenti-Cas9-2A-Blasticidin vector (Addgene, #73310)[9] by replacing the Cas9 sequence with the sequence of H2B-miRFP703 (Addgene, #80001) using the NEBuilder HiFi DNA Assembly kit. The resulting vector was used to make lentiviral particles, as previously described[10]. Briefly, human embryonic kidney 293T (HEK 293T; ECACC 12022001) cells were transfected using X-TremeGENE 9 (Roche) with plasmids pLenti-H2B-miRFP703-2A-Blasticidin, psPAX2 (packaging plasmid; Addgene, #12260) and pMD2.G (VSV-G envelop plasmid; Addgene, # 12259) to generate VSV-G-coated lentiviral particles. Both psPAX2 and pMD2.G were a gift from Didier Trono. At 24 h post transfection, the medium was replaced by DMEM supplemented with 1.1 g/100mL bovine serum albumin. At 72 h post transfection, supernatant containing lentiviral particles was collected and used to transduce Vero E6 cells in the presence of polybrene (8 μg/mL). After 24 h, medium was removed, and cells were treated with blasticidin (Thermo Fisher Scientific) at a concentration of 5 μg/mL. Surviving cells were maintained in MEM with 10% FBS and 5 μg/mL blasticidin for further studies.

A549-NIR originating from A549 (human lung carcinoma; ATCC CCL-185) and constitutively expressing miRFP cells were generated accordingly.

Characterization of DENV-2 Reporter Viruses

To develop an image-based antiviral assay with multiple reporter viruses, we first evaluated to what extent the choice of fluorescent protein (FP) may impact viral replication fitness. All FPs considered are derivatives of Green Fluorescent Protein (GFP) or its orthologs such as mRFP1 (monomeric Red Fluorescent Protein 1) sharing a high degree of sequence similarity[11,12]. Theoretically, the resulting recombinant viruses can be expected to have very similar characteristics, except for the excitation and emission spectra of their fluorescent signals. For direct head-to-head comparison, a series of DENV-2 reporter viruses was generated encoding for an array of five different FPs (DENV-2/X-FPs), namely DENV-2/mAzurite (blue), DENV-2/eGFP (green), DENV-2/mCitrine (yellow), DENV-2/mCherry (red) and DENV-2/mMaroon (far-red) (Supplementary Fig. 1a). All constructs yielded viable virus variants that were, however, slightly attenuated, compared to wild-type DENV-2 (DENV-2/WT); showing smaller plaques on BHK21J cells than DENV-2/WT (Supplementary Fig. 1b)[1].

High-content imaging (HCI; Arrayscan XTI) of infected V-NIR cells was used to investigate the brightness and possible spectral interference of fluorescent signals from different DENV-2/X-FP variants. Custom channels with specific settings for excitation wavelength, dichroic

mirror placement, emission filter and exposure time to detect V-NIR and cells infected by each DENV-2/X-FP are shown in Supplementary Table 1 as optimized for detection and for minimal signal interference between channels. Here, mAzurite signals could only be detected in the specific Azurite channel, yet without issues of emission signal bleed-through into other channels. However, strong mutual interference was observed for both eGFP and mCitrine due to obvious spectral overlap and instrumental limits (properties of available filters). We also observed strong bleed-through signals of mCherry into the Maroon channel, while no bleed-through emission of mMaroon was detected in the Cherry channel. However, signals of mMaroon were exceptionally weak, requiring long exposure times for detection (Supplementary Fig. 1c; Supplementary Table 1).

Further, we investigated the relative virus replication competence on Vero E6 cells at different multiplicities of infection (MOI). First, a low MOI of 0.01 was used to compare the viral growth kinetics of DENV-2/WT, DENV-2/mAzurite, DENV-2/eGFP, DENV-2/mCitrine, and DENV-2/mCherry. Generally, all DENV-2/X-FPs showed very similar, yet slightly lower growth than DENV-2/WT, with no significant differences regarding virus yields among the different DENV-2/X-FP variants (Supplementary Fig. 2a, 0.01 MOI). As we have shown previously, even at a 10-fold higher MOI of 0.1, no significant difference between DENV-2/WT and DENV-2/mCherry were observed up to 4 dpi[1].

We then focused on virus yields of FP-tagged variants, representing four main colors of the available spectrum: red (DENV-2/mCherry), blue (DENV-2/mAzurite), green/yellow (DENV-2/mCitrine), and far-red (DENV-2/mMaroon). In this direct comparison, DENV-2/mMaroon was outcompeted by the other DENV-2/X-FPs with significantly lower virus yields (Supplementary Fig. 2b, 0.1 MOI). Finally, we checked the viability of V-NIR cells (Supplementary Fig. 2c, 0.1 MOI) as function of infection (Supplementary Fig. 2d, 0.1 MOI), clearly demonstrating that cell counts were not skewed due to virus-induced CPE at least until 4 dpi (no major loss of cells). Under these conditions of preserved cell viable, the infection rates of DENV-2/mMaroon were again lower than for the other DENV-2/X-FPs (Supplementary Fig. 2d).

In summary, all recombinant reporter DENV-2 viruses remained fully replication competent irrespectively of the FP variant used, with an almost uncompromised fitness compared to DENV-2/WT; with possible exception DENV-2/mMaroon. This suggests that basically any major spectral FP variants should be equally amenable for HCI assay development using fluorescent reporter viruses.

Characterization of YF17D/mCherry and JEV/eGFP reporter viruses

The construction and use of YF17D/mCherry and JEV/eGFP has been described before [4,8,13]. Either reporter virus was compared to its parental virus by two means; (***i***) by plaque assay on BHK-21J cells and (*ii*) by assessment of viral growth kinetics on Vero E6 cells and titration of respective virus yields using MTS [3-(4,5-dimethylthiazol-2-yl)-5-(3-carboxymethoxyphenyl)-2-(4-sulfophenyl)-2H-tetrazolium] dye conversion (Promega) as read-out for virus-induced CPE to calculate respective median tissue culture infectious doses ($TCID_{50}$)[8].

Both reporter viruses YF17D/mCherry and JEV/eGFP remained fully replication competent, able to cause virus lysis plaques and CPE. Likewise, similar to the DENV-2/X-FP constructs, both reporter viruses showed a smaller plaque size (Fig. 1f), slightly reduced viral growth kinetics, and lower virus yields compared to their corresponding parent viruses (Supplementary Fig. 3). Notably, in either case, reporter virus titers as scored by fluorescence microscopy resembled those $TCID_{50}$ titers of their parent viruses obtained by CPE scoring.

Supplementary Table

Supplementary Table 1. Optical channel settings for multicolor assay on a 7 color-engine widefield Arrayscan XTI high-content imager (Thermo Fisher Scientific)

Custom channels	Excitation (nm)	Dichroic mirror	Emission filter	Average exposure time (ms)
V-NIR	650-13	BGRFRN	BGRFRN	600
Azurite	386-23	BGRFRN	BGRFRN	20
GFP	485-20	BGS	Y	60
Citrine	485-20	Y	Y	18
Cherry	560-25	BGRFRN	BGRFRN	3
Maroon	560-25	BGRFRN	NIR	600

Supplementary Figures

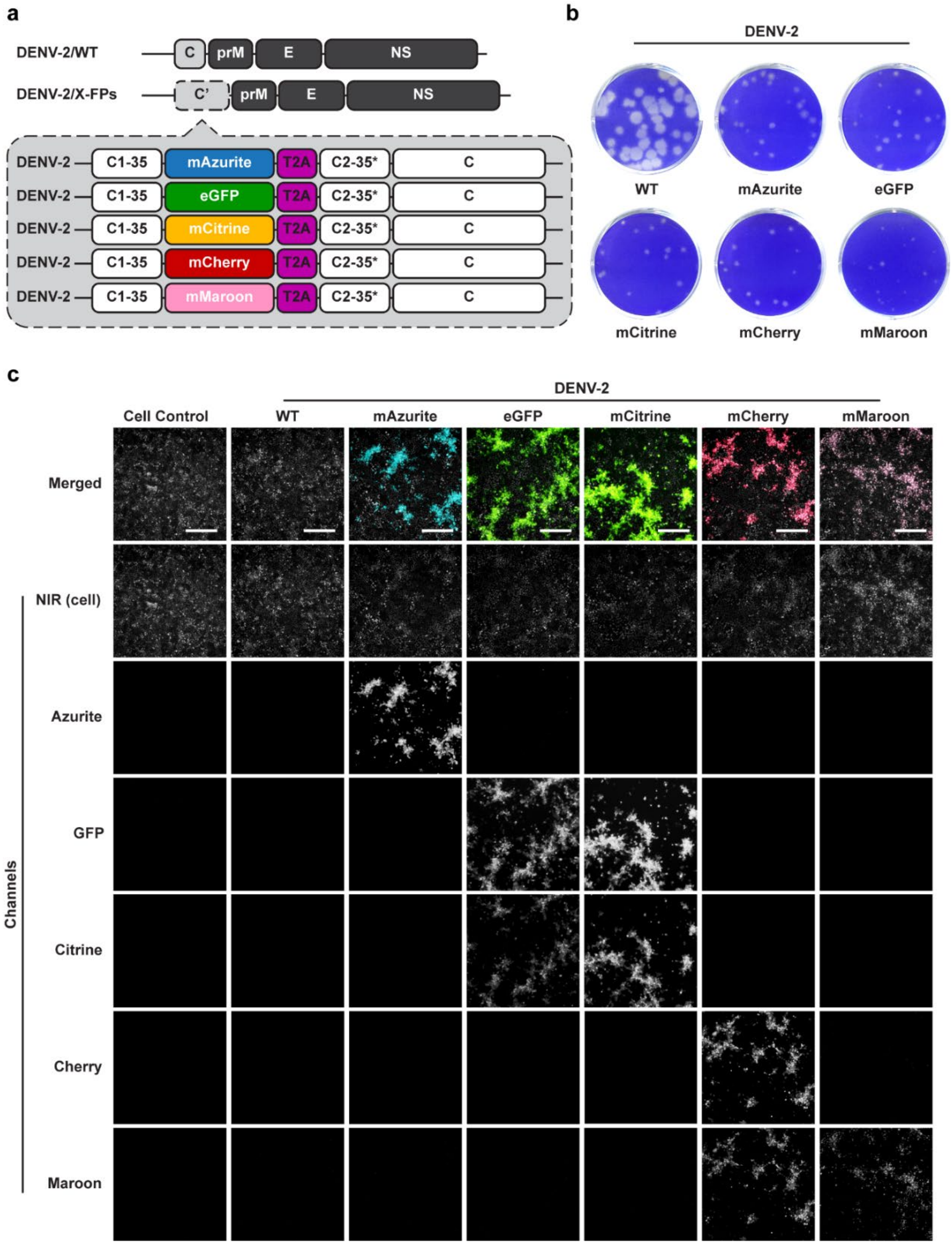

Supplementary Fig. 1 Characteristics of dengue type 2 reporter viruses (DENV-2/X-FPs) (related to Fig. 1). a) Construct design of DENV-2 reporter viruses[1]. Individual reporter viruses are identical with only the reporter gene (encoding for spectral variants of fluorescent protein) replaced by different fluorescent proteins (FPs) as shown. **b) Plaque phenotype of DENV-2/WT (no reporter gene inserted) and DENV-2 reporter viruses (DENV-2/X-FPs). c HCI readout of multicolor DENV-2 reporter viruses.** V-NIR cells were infected with DENV-2/WT or DENV-2/X-FPs at an MOI of 0.1. Fluorescent images taken on live infected cells by HCI at 5 dpi. Scale bar: 255 µm.

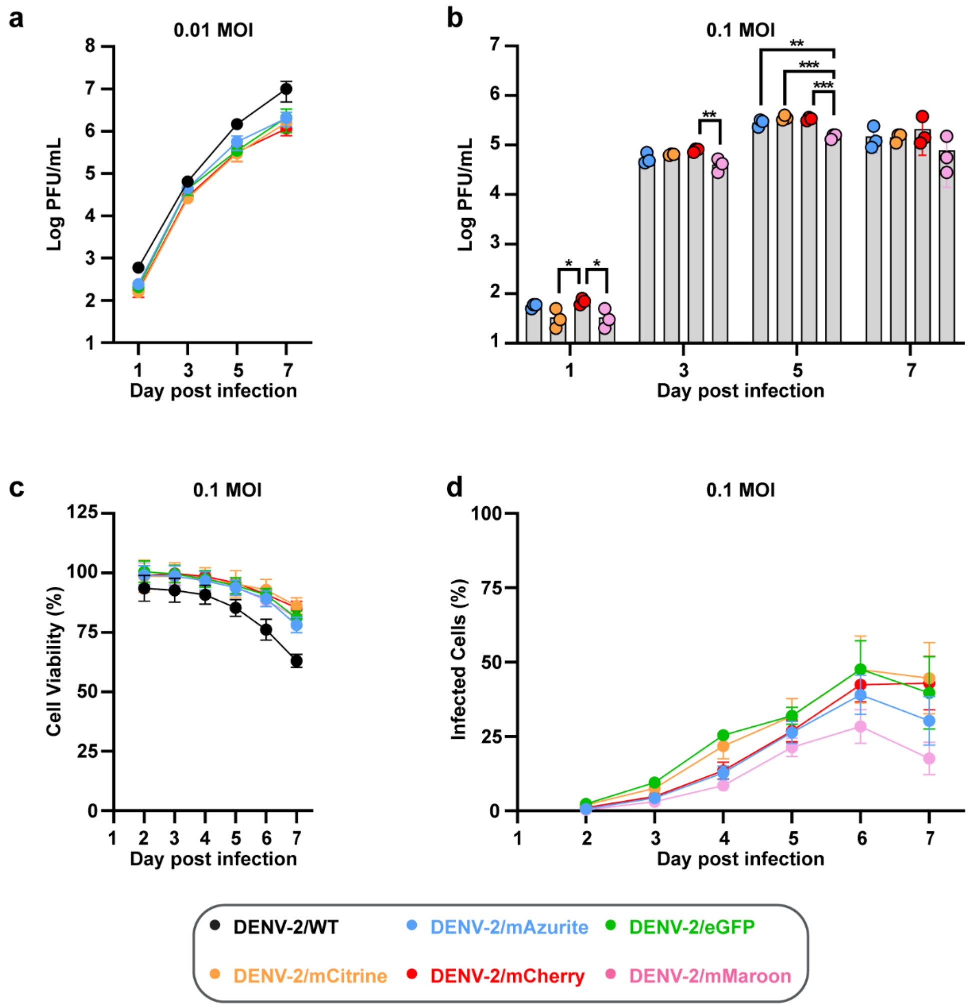

Supplementary Fig. 2 Growth kinetics of dengue type 2 reporter viruses (DENV-2/X-FPs) (related to Supplementary Fig. 1). a) Replication kinetics of recombinant DENV-2 reporter viruses in comparison to wild-type DENV-2 NGC parental strain. Vero E6 cells were infected with DENV-2/WT, DENV-2/mAzurite, DENV-2/eGFP, DENV-2/mCitrine, or DENV-2/mCherry at an MOI of 0.01. Supernatants of the infected cells were harvested at 1, 3, 5 and 7 dpi. Virus titers determined by plaque assays on BHK21J cells. Data presented means ± SD of n=3 independent experiments. **b) Direct comparison of virus yields for different DENV-2/X-FPs.** Vero E6 cells were infected with DENV-2/mAzurite, DENV-2/mCitrine, DENV-2/mCherry, or DENV-2/mMaroon at an MOI of 0.1. Supernatants harvested at 1, 3, 5, and 7 dpi and virus yields determined by plaque assays. The data presented means ± SD of n=3 independent experiments. **c) HCI readout of cell survival rate** and **d) virus infectivity over time.** V-NIR cells infected at an MOI of 0.1. From 2 to 7 dpi, the indicated fluorescent signals were detected by HCI using filter settings as specified in Supplementary Table 1. Cell viability (%) defined as number of NIR positive cells in DENV-2 reporter virus infected wells versus number in uninfected wells; virus infectivity defined as number of cells with respective fluorescent signal versus NIR positive cells in the same well. Data presented means ± SD of n=6 independent experiments. P values calculated by one-way ANOVA with Tukey's post hoc

test. P < 0.05 considered statistically significant (* = p< 0.05; ** = p<0.01; *** = p<0.001). Source data are provided as a Source Data file.

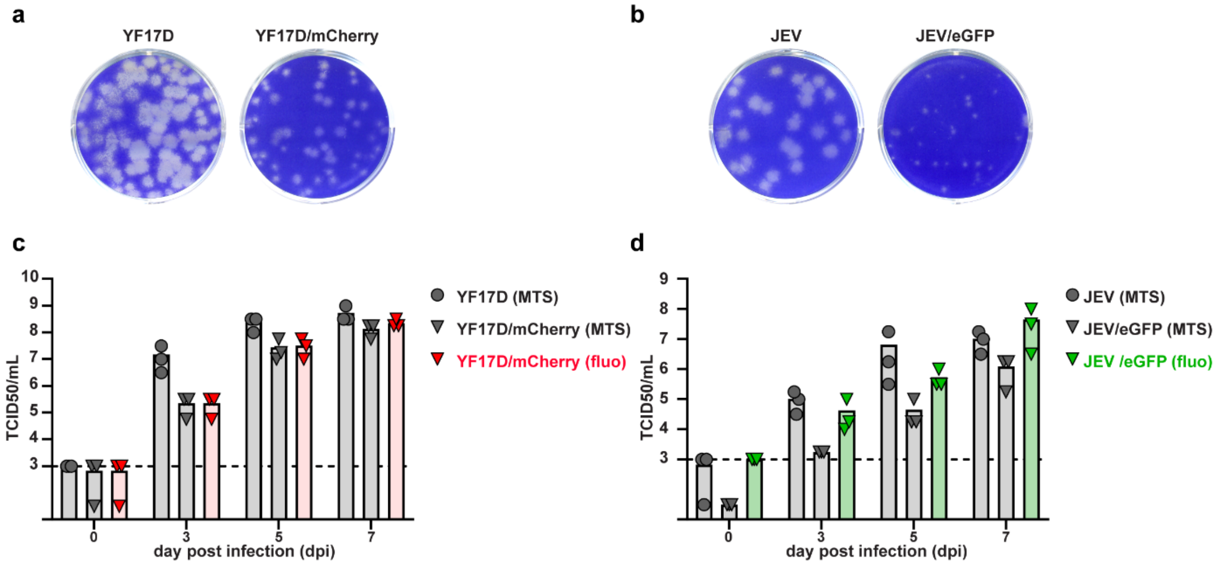

Supplementary Fig. 3 (related to Fig. 1f). Growth kinetics of YF17D- and JEV-derived reporter viruses and their parental viruses. Plaque assay for the titer determination of working virus stocks. **a)** Plaque phenotypes of YF17D and YF17D/mCherry. **b)** Plaque phenotypes of JEV and JEV/eGFP. **c-d)** Vero E6 cells were infected with the respective viruses at an MOI of 0.01. Supernatants of the infected cells were collected at 0, 3, 5 and 7 dpi. Infectious virus titers were quantified as median tissue culture infectious dose ($TCID_{50}$/mL) scoring for CPE by MTS dye conversion, or by fluorescent signals on infected BHK-21J cells. Each individual repeat (n=3) shown, and average depicted as columns. Source data are provided as a Source Data file.

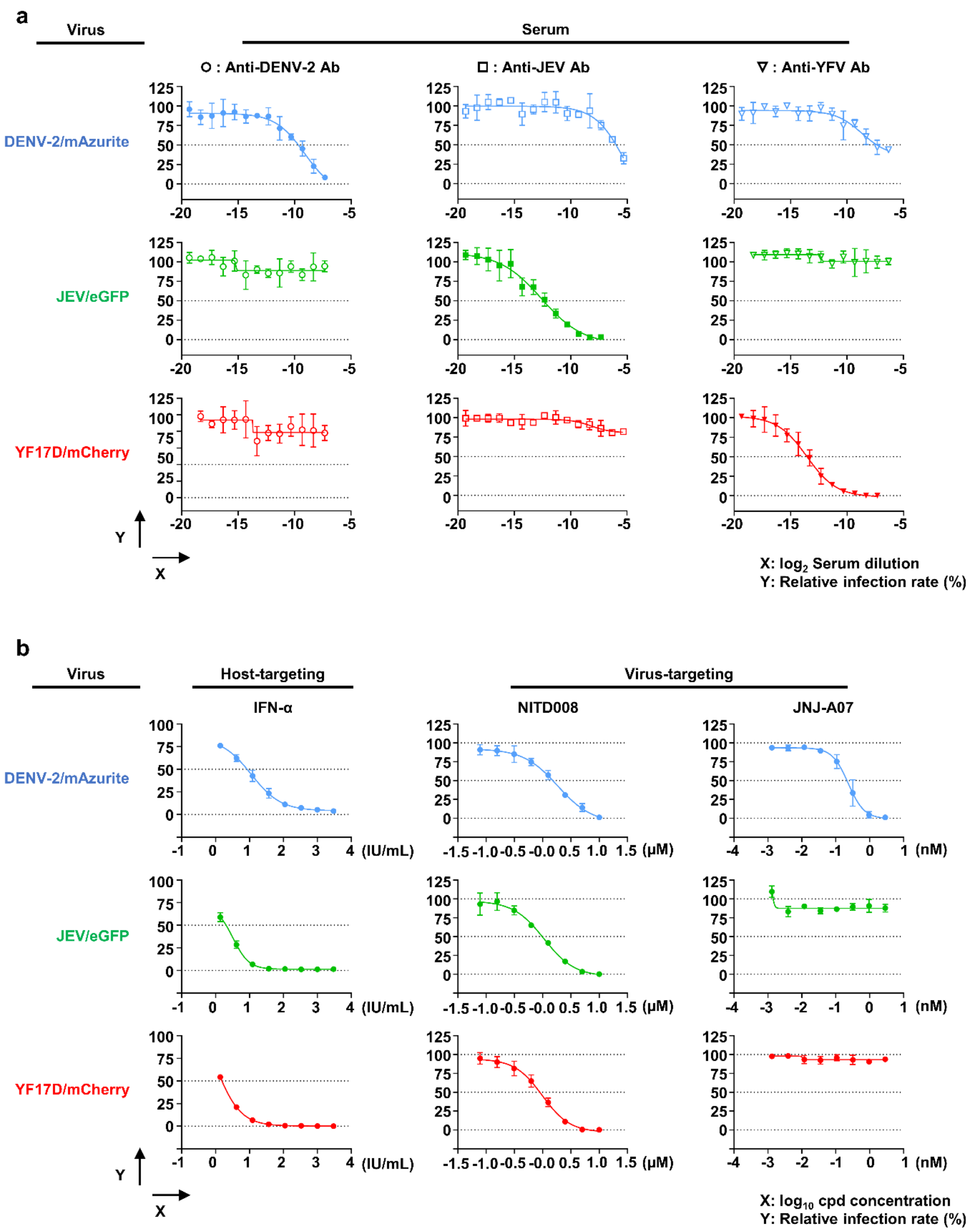

Supplementary Fig. 4 (related to Fig. 3). Single virus assay for SNT or antiviral assays. a) Virus (DENV-2/mAzurite, JEV/eGFP, or YF17D/mCherry) was preincubated with serial dilutions of sera containing anti-DENV-2 (circle), anti-JEV (square), or anti-YFV (triangle) antibodies (Ab), respectively, and then used to infect V-NIR cells at an MOI of 0.5 for DENV-2/mAzurite, 0.5 for JEV/eGFP, and 0.2 for YF17D/mCherry. **b)** V-NIR cells were infected with DENV-2/mAzurite (MOI=0.5), JEV/eGFP (MOI=0.5), or YF17D/mCherry (MOI=0.2), and treated with serial dilutions concentrations of IFN-α, NITD008 or JNJ-A07. Experimental details described in materials and methods (SNT and Antiviral assay). At 3 dpi, cells expressing specified fluorescence signals (infected cells) were detected and quantified by HCI. Representative neutralizing curves (a) or effect curves (b) were plotted using GraphPad Prism 8 and nonlinear regression. Source data are provided as a Source Data file.

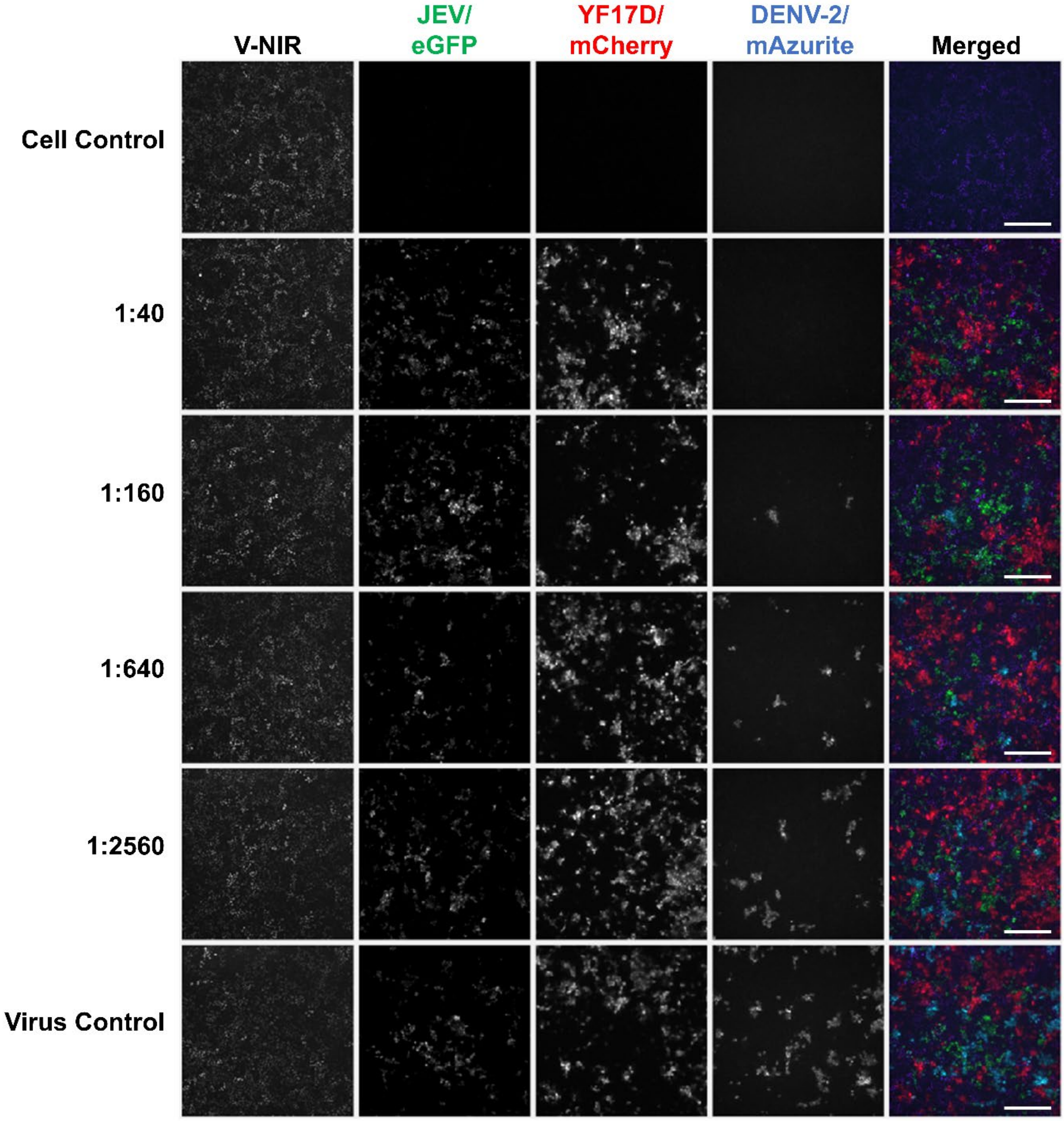

Supplementary Fig. 5 (related to Fig. 3a, b). Fluorescent images of infected V-NIR cells in multi-orthoflavi serum neutralizing assay (SNT) using anti-DENV-2 serum. DENV-2/mAzurite (blue, 0.5 MOI), JEV/eGFP (green, 0.5 MOI) and YF17D/mCherry (red, 0.2 MOI) were preincubated with two-time serial dilutions of antiserum containing DENV-2 neutralizing antibodies for 75 min prior to infection of V-NIR cells. Fluorescent images taken on live infected V-NIR cells by HCI at 3 dpi. The images presented for increasing dilutions from 1:40 to 1:2560, cell control (no virus added, top) and virus control (no serum added, bottom). Scale bar: 255 μm.

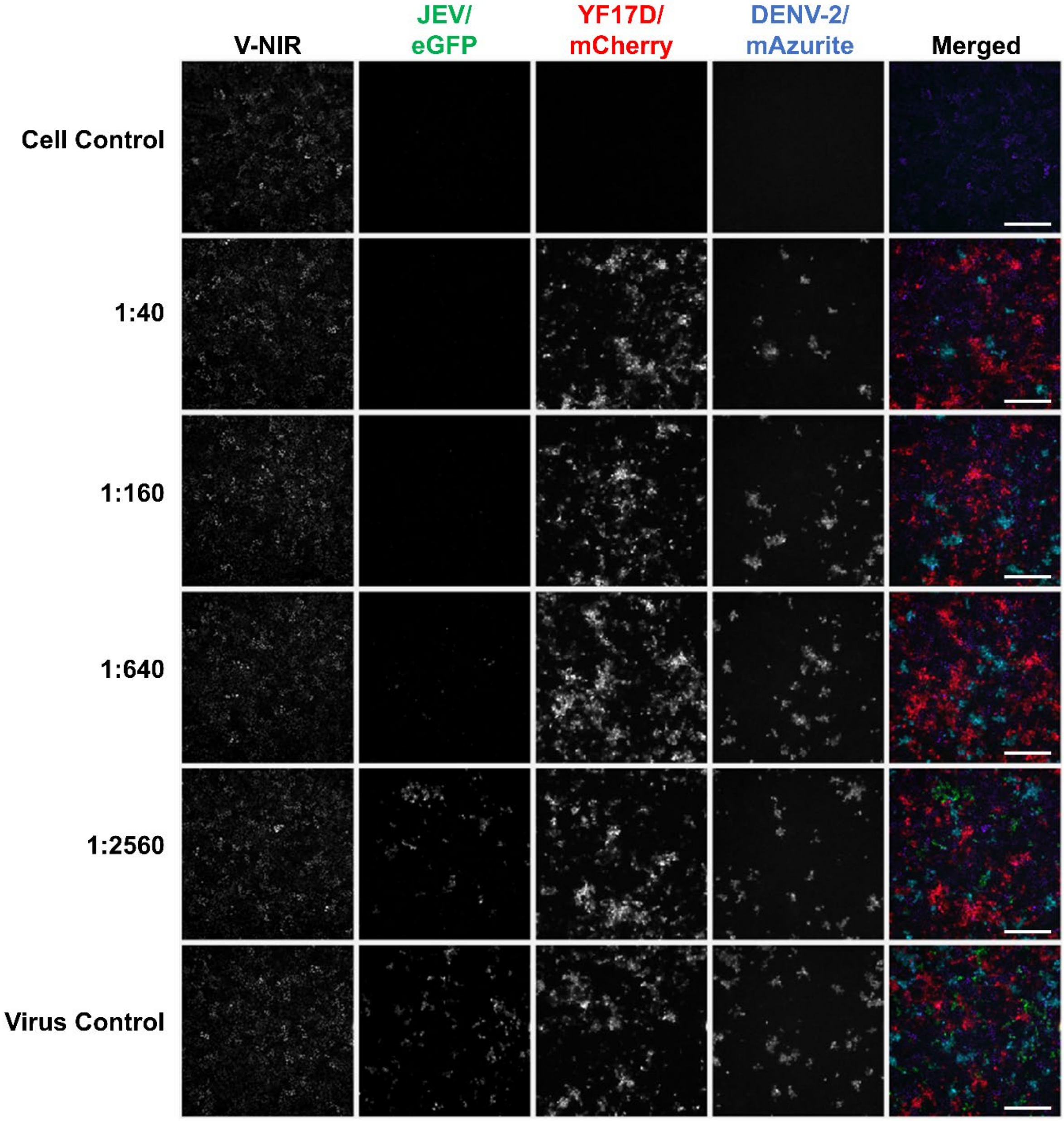

Supplementary Fig. 6 (related to Fig. 3a, b). Fluorescent images of infected V-NIR cells in multi-orthoflavi serum neutralizing assay (SNT) using anti-JEV serum. DENV-2/mAzurite (blue, 0.5 MOI), JEV/eGFP (green, 0.5 MOI) and YF17D/ mCherry (red, 0.2 MOI) were preincubated with two-time serial dilutions of antiserum containing JEV neutralizing antibodies for 75 min prior to infection of V-NIR cells. Fluorescent images taken on live infected V-NIR cells by HCI at 3 dpi. The images presented for increasing dilutions from 1:40 to 1:2560, cell control (no virus added, top) and virus control (no serum added, bottom). Scale bar: 255 µm.

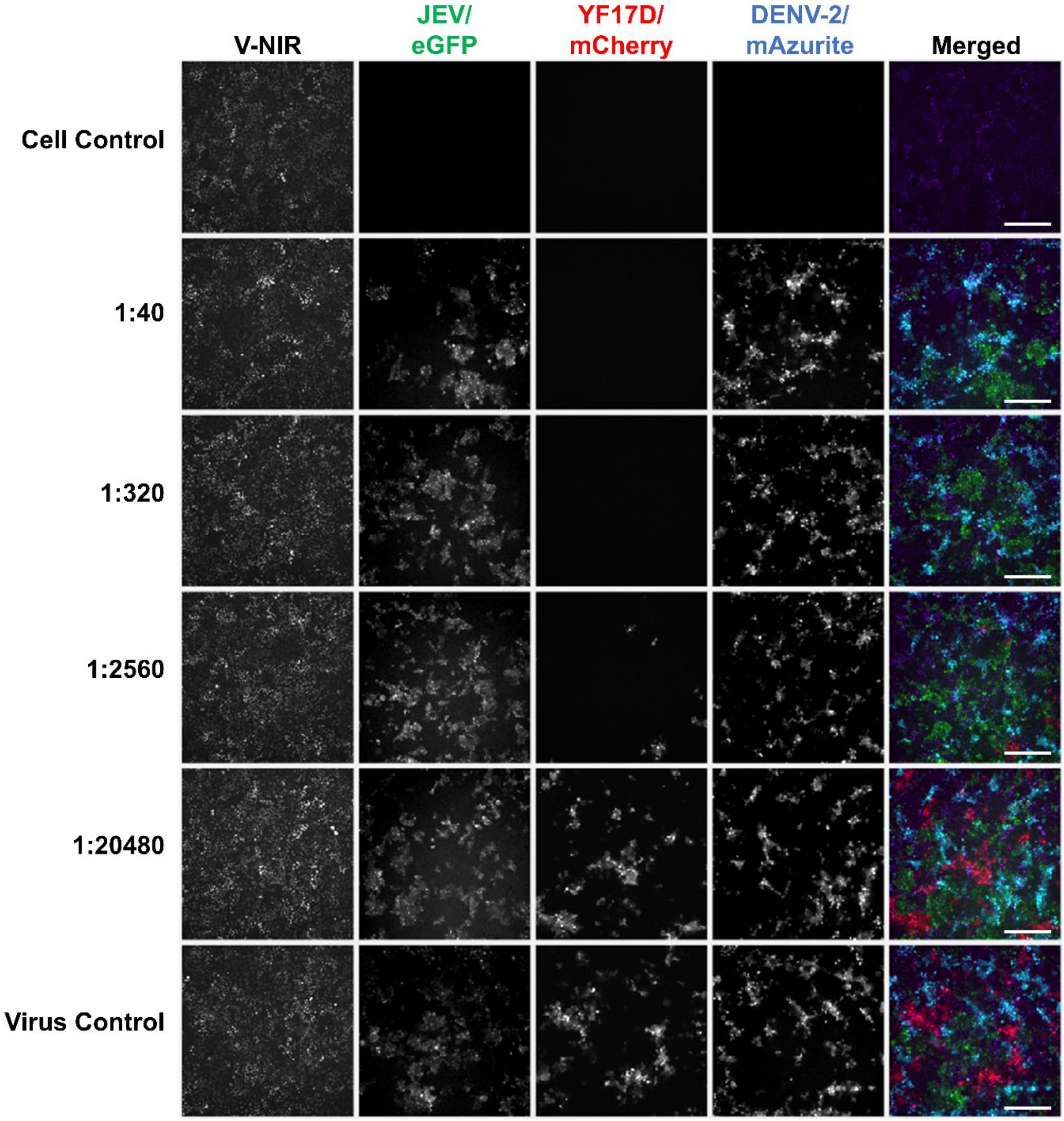

Supplementary Fig. 7 (related to Fig. 3a, b). Fluorescent images of infected V-NIR cells in multi-orthoflavi serum neutralizing assay (SNT) using anti-YFV serum. DENV-2/mAzurite (blue, 0.5 MOI), JEV/eGFP (green, 0.5 MOI) and YF17D/ mCherry (red, 0.2 MOI) were preincubated with two-time serial dilutions of antiserum containing YFV neutralizing antibodies for 75 min prior to infection of V-NIR cells. Fluorescent images taken on live infected V-NIR cells by HCI at 3 dpi. The images presented for increasing dilutions from 1:40 to 1:20480, cell control (no virus added, top) and virus control (no serum added, bottom). Scale bar: 255 μm.

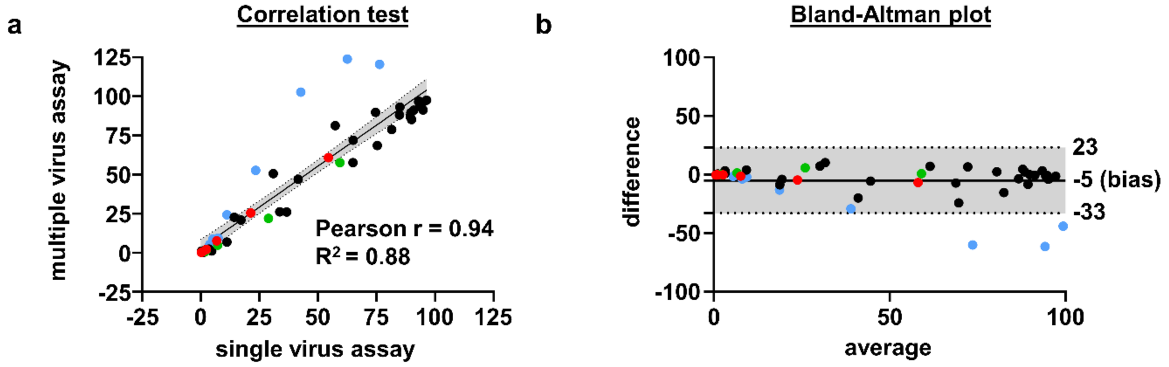

Supplementary Fig. 8 (related to Fig. 3f). Correlation analysis (a) and Blant-Altman test (b). Data displayed exactly as Fig. 3f yet data pairs corresponding to IFN-α treated conditions are highlighted in color (DENV-2/mAzurite, blue; JEV/eGFP, green; YF17D/mCherry, red). **a)** Due to a competitive advantage of DENV-2 over JEV and YFV in presence of IFN-α, the overall correlation (Pearson coefficient r = 0.94; coefficient of determinations R^2 = 0.88) is skewed in the multiplex-virus assay (blue circles with y-values > 100% outside of 95% CI) during that particular treatment. Values on both x- and y-axis displaying relative infection rates in %. **b)** Bland-Altman analysis with same outliers for DENV-2/mAzurite as in (a) failing to fall within limits of agreements. The data presented means of n=3 independent experiments.

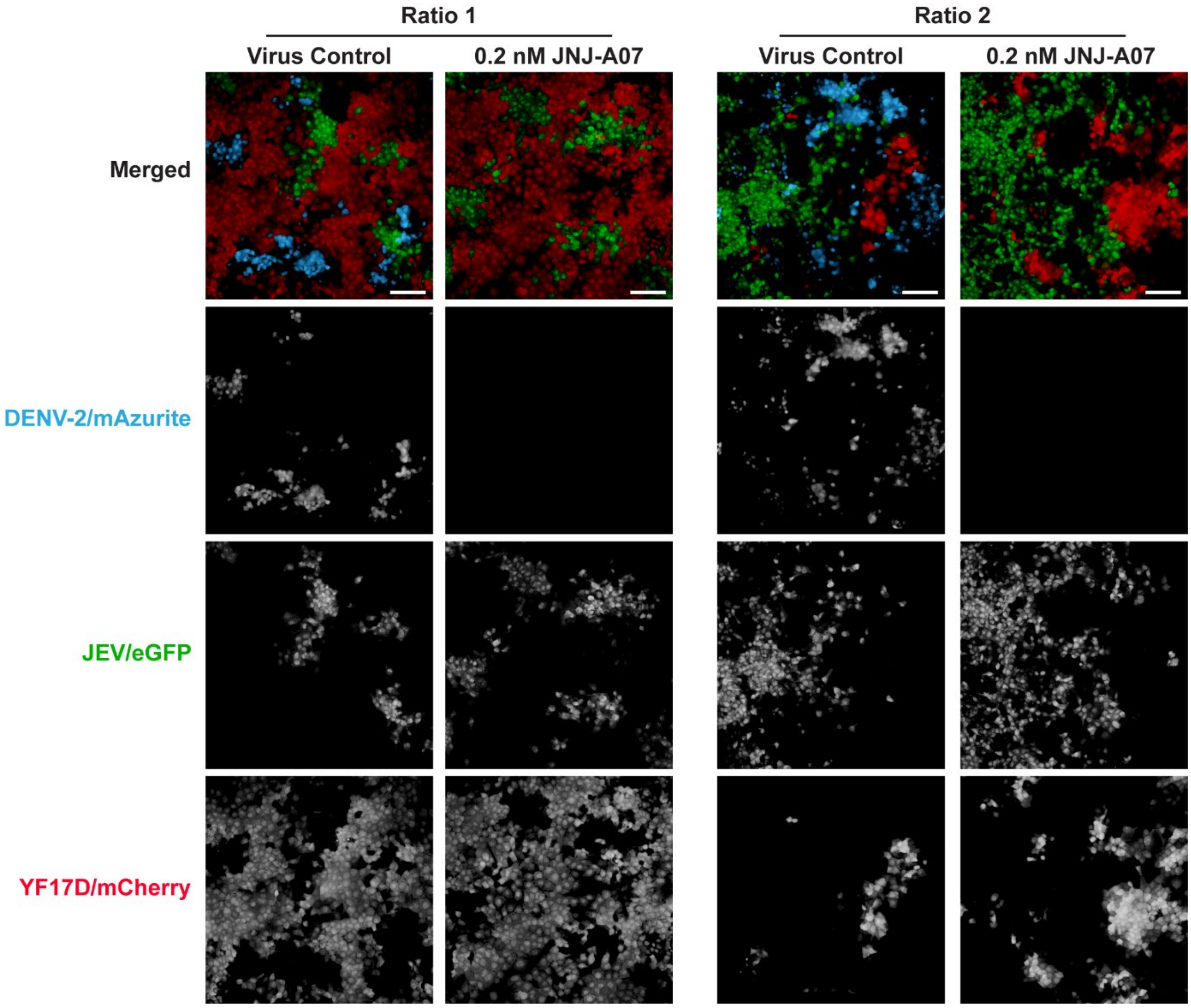

Supplementary Fig. 9. Multiplex flavivirus infections on Huh-7 cells. Huh-7 cells were infected with the virus mixture only or virus-compound mixture as indicated as Ratio 1 or Ratio 2, with the final concentration of 0.2 nM JNJ-A07 for the virus-compound mixture. Ratio 1 contains 0.1 MOI for DENV-2/mAzurite, 0.1 MOI for JEV/eGFP, and 0.01 MOI for YFV/mCherry, while Ratio 2 contains 0.5 MOI for DENV-2/mAzurite, 0.4 MOI for JEV/eGFP, and 0.1 MOI for YFV/mCherry. For the condition of Ratio 1, the cells were fixed at 3 dpi, while for Ratio 2, the cells were fixed at 2 dpi. Cells expressing specified fluorescence signals (infected cells) were detected by HCI. Images were acquired using an Operetta CLS High Content Imaging and Analysis system (Revvity, Waltham, Massachusetts, USA). Scale bar: 100 µm.

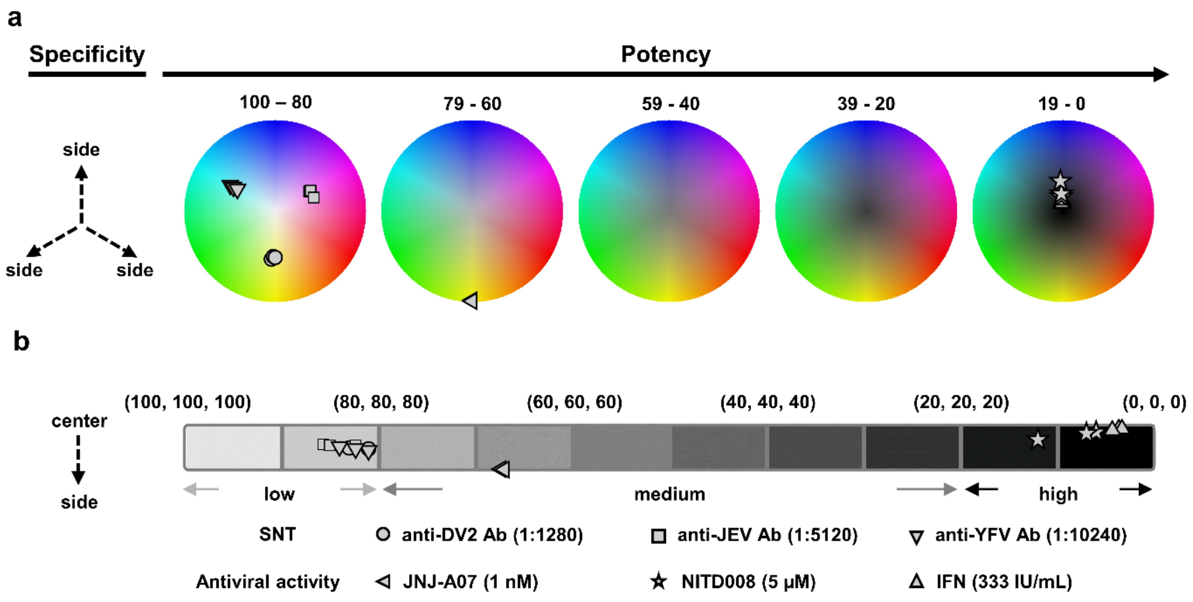

Supplementary Fig. 10 (related to Fig. 3). Selected inhibitor profiles on the virus RGB model. RGB color mixing was used to convert SNT_{50} and EC_{90} values (as obtained in Fig. 3; see also Table 1 and 2) into specific color codes. Based on the RGB concept, the codes can be deconvoluted on a series of RGB palettes **(a)** and a white-black scale **(b)**, visualizing the full profile of specificity and potency of that particular treatment.

Supplementary References

1. Li, L.-H., *et al.* A dengue type 2 reporter virus assay amenable to high-throughput screening. *Antiviral Research* **183**, 104929 (2020).
2. Gualano, R.C., Pryor, M.J., Cauchi, M.R., Wright, P.J. & Davidson, A.D. Identification of a major determinant of mouse neurovirulence of dengue virus type 2 using stably cloned genomic-length cDNA. *Journal of general virology* **79**, 437-446 (1998).
3. Dallmeier, K. & Neyts, J. Simple and inexpensive three-step rapid amplification of cDNA 5′ ends using 5′ phosphorylated primers. *Analytical biochemistry* **434**, 1-3 (2013).
4. Kum, D.B., *et al.* A yellow fever–Zika chimeric virus vaccine candidate protects against Zika infection and congenital malformations in mice. *npj Vaccines* **3**, 56 (2018).
5. Mena, M.A., Treynor, T.P., Mayo, S.L. & Daugherty, P.S. Blue fluorescent proteins with enhanced brightness and photostability from a structurally targeted library. *Nature biotechnology* **24**, 1569-1571 (2006).
6. Griesbeck, O., Baird, G.S., Campbell, R.E., Zacharias, D.A. & Tsien, R.Y. Reducing the environmental sensitivity of yellow fluorescent protein: mechanism and applications. *Journal of biological chemistry* **276**, 29188-29194 (2001).
7. Gietz, R.D. & Schiestl, R.H. Frozen competent yeast cells that can be transformed with high efficiency using the LiAc/SS carrier DNA/PEG method. *Nature protocols* **2**, 1-4 (2007).
8. Mishra, N., *et al.* A chimeric Japanese encephalitis vaccine protects against lethal yellow fever virus infection without inducing neutralizing antibodies. *MBio* **11**, e02494-02419 (2020).
9. Hart, T., *et al.* High-resolution CRISPR screens reveal fitness genes and genotype-specific cancer liabilities. *Cell* **163**, 1515-1526 (2015).
10. Baggen, J., *et al.* Genome-wide CRISPR screening identifies TMEM106B as a proviral host factor for SARS-CoV-2. *Nature Genetics* **53**, 435-444 (2021).
11. Campbell, R.E., *et al.* A monomeric red fluorescent protein. *Proceedings of the National Academy of Sciences* **99**, 7877-7882 (2002).
12. Shu, X., Shaner, N.C., Yarbrough, C.A., Tsien, R.Y. & Remington, S.J. Novel chromophores and buried charges control color in mFruits. *Biochemistry* **45**, 9639-9647 (2006).
13. Rasulova, M., *et al.* A high-throughput yellow fever neutralization assay. *Microbiology Spectrum* **10**, e02548-02521 (2022).
