# Supplementary material for: Multiplexed multicolor antiviral assay amenable for high-throughput research
Source: Nat Commun. 2024 Jan 2;15:42. doi: 10.1038/s41467-023-44339-z (PMC10761739; doi:10.1038/s41467-023-44339-z)
Supplement: Supplementary file 3 — Description of Additional Supplementary Files [file 41467_2023_44339_MOESM3_ESM.pdf]

Description of Additional Supplementary Files

Supplementary Movie Legends:

Supplementary Movie 1: 3D cube of the RGB model representing range of possible antiviral activities obtained by multiplex-virus assay and colors tied to the three Cartesian coordinates (related to Fig. 5).

Supplementary Movie 2: Examples of possible antiviral activities obtained by multiplex-virus assay and colors tied to the respective coordinates in the 3D RGB model. Circle – full (100%) inhibition of DENV-2/mAzurite, i.e., RGB coordinate (100, 100, 0); square – full inhibition of DENV-2/mAzurite plus 50% inhibition of JEV/eGFP, i.e., RGB coordinate (100, 50, 0) (related to Fig. 5).

Supplementary Movie 3: Animation for a more comprehensive illustration and visualization of the 3D model, with a specific focus on explaining the origins of both the RGB palettes and the WB projections (related to Fig. 5).
