# Supplementary material for: Multiplexed multicolor antiviral assay amenable for high-throughput research
Source: Nat Commun. 2024 Jan 2;15:42. doi: 10.1038/s41467-023-44339-z (PMC10761739; doi:10.1038/s41467-023-44339-z)
Supplement: Supplementary file 7 — Reporting Summary [file 41467_2023_44339_MOESM7_ESM.pdf]

nature portfolio

Corresponding author(s): Kai Dallmeier

Last updated by author(s): Nov 10, 2023

Reporting Summary

Nature Portfolio wishes to improve the reproducibility of the work that we publish. This form provides structure for consistency and transparency in reporting. For further information on Nature Portfolio policies, see our Editorial Policies and the Editorial Policy Checklist.

Statistics

For all statistical analyses, confirm that the following items are present in the figure legend, table legend, main text, or Methods section.

n/a	Confirmed	
☐	☒	The exact sample size (*n*) for each experimental group/condition, given as a discrete number and unit of measurement
☐	☒	A statement on whether measurements were taken from distinct samples or whether the same sample was measured repeatedly
☐	☒	The statistical test(s) used AND whether they are one- or two-sided<br>*Only common tests should be described solely by name; describe more complex techniques in the Methods section.*
☒	☐	A description of all covariates tested
☐	☒	A description of any assumptions or corrections, such as tests of normality and adjustment for multiple comparisons
☐	☒	A full description of the statistical parameters including central tendency (e.g. means) or other basic estimates (e.g. regression coefficient) AND variation (e.g. standard deviation) or associated estimates of uncertainty (e.g. confidence intervals)
☐	☒	For null hypothesis testing, the test statistic (e.g. *F*, *t*, *r*) with confidence intervals, effect sizes, degrees of freedom and *P* value noted<br>*Give P values as exact values whenever suitable.*
☒	☐	For Bayesian analysis, information on the choice of priors and Markov chain Monte Carlo settings
☒	☐	For hierarchical and complex designs, identification of the appropriate level for tests and full reporting of outcomes
☐	☒	Estimates of effect sizes (e.g. Cohen's *d*, Pearson's *r*), indicating how they were calculated

Our web collection on statistics for biologists contains articles on many of the points above.

Software and code

Policy information about availability of computer code

Data collection	High content image collection performed by using a 7 color-engine widefield high content screening platform (Arrayscan XTI, Thermo Fisher Scientific). Except for Huh-7 cells (Supplementary Fig. 9), images were acquired using an Operetta CLS High Content Imaging and Analysis system (Revvity, Waltham, Massachusetts, USA).
Data analysis	GraphPad Prism (version 10.0.0 or earlier version) was used for all statistical evaluations.<br>The RGB model was executed using MATLAB (Version 2017b, The MathWorks, Inc., Natick, Massachusetts) with an in-house developed software.<br>The code written for automated data analysis and visualization is deposited at GitHub and can be accessed through GitHub (https://github.com/YunAnGitHub/RGB_Virus_Model). This package has also been deposited at Zenodo: https://zenodo.org/records/10113251

For manuscripts utilizing custom algorithms or software that are central to the research but not yet described in published literature, software must be made available to editors and reviewers. We strongly encourage code deposition in a community repository (e.g. GitHub). See the Nature Portfolio guidelines for submitting code & software for further information.

Data

Policy information about availability of data

All manuscripts must include a data availability statement. This statement should provide the following information, where applicable:

- Accession codes, unique identifiers, or web links for publicly available datasets
- A description of any restrictions on data availability
- For clinical datasets or third party data, please ensure that the statement adheres to our policy

All relevant data of this study are available in the paper and its supplementary materials. Source data are provided with this paper.

Research involving human participants, their data, or biological material

Policy information about studies with human participants or human data. See also policy information about sex, gender (identity/presentation), and sexual orientation and race, ethnicity and racism.

Reporting on sex and gender	not applicable
Reporting on race, ethnicity, or other socially relevant groupings	not applicable
Population characteristics	not applicable
Recruitment	not applicable
Ethics oversight	not applicable

Note that full information on the approval of the study protocol must also be provided in the manuscript.

Field-specific reporting

Please select the one below that is the best fit for your research. If you are not sure, read the appropriate sections before making your selection.

☒ Life sciences ☐ Behavioural & social sciences ☐ Ecological, evolutionary & environmental sciences

For a reference copy of the document with all sections, see nature.com/documents/nr-reporting-summary-flat.pdf

Life sciences study design

All studies must disclose on these points even when the disclosure is negative.

Sample size	No statistical method was used to predetermine sample size.<br>Instead, at least n=3 biological repeats were performed as from a-priori routine.
Data exclusions	No data were excluded from analysis.
Replication	Unless explicitly specified in the figure legends or outlined below, all experiments were repeated three times, and data are represented as means ± SD from 3 independent biological repeats. For kinetics of individual reporter flavivirus infections (Fig. 1e), data are represented as means ± SD from 4 independent biological repeats . For assessing assay robustness (Fig. 2b), Z' values were determined from six replicates (n=6) at both 3 dpi and 4 dpi. In the case of multiplex infections presented in Fig. 4, two independent experiments were performed for each combination, except for the combination of DENV-2/mAzurite, PIV1/eGFP, and CHIKV/mCherry, which was performed only once. In the pilot-scale library screen (Fig. 6a-c), five reference plates were used. All representative micrographs presented in this study were from experiments which repeated at least three times and yield similar results.
Randomization	Experiments in this study were not randomized as a fixed layout was used on each scanning plate with cells assigned to either the predetermined experimental or control groups.
Blinding	No specific group allocations were involved in this study, the concept of researcher blinding was not applicable. Notably, the investigators did not receive information regarding the compound library used in the HTS and the identity of the respective small molecules remained undisclosed throughout the study.

Reporting for specific materials, systems and methods

We require information from authors about some types of materials, experimental systems and methods used in many studies. Here, indicate whether each material, system or method listed is relevant to your study. If you are not sure if a list item applies to your research, read the appropriate section before selecting a response.

Materials & experimental systems

n/a	Involved in the study	
☐	☒	Antibodies
☐	☒	Eukaryotic cell lines
☒	☐	Palaeontology and archaeology
☒	☐	Animals and other organisms
☒	☐	Clinical data
☒	☐	Dual use research of concern
☒	☐	Plants

Methods

n/a	Involved in the study	
☒	☐	ChIP-seq
☒	☐	Flow cytometry
☒	☐	MRI-based neuroimaging

Antibodies

Antibodies used	polyclonal sera from mice immunized using either YF17D, JE SA14-14-2, or DV2 NGC live-attenuated viruses
Validation	Rasulova, M. et al. A high-throughput yellow fever neutralization assay. Microbiology Spectrum 10, e02548-02521 (2022).

Eukaryotic cell lines

Policy information about cell lines and Sex and Gender in Research

Cell line source(s)	BHK-21J cells (Baby hamster kidney cells; Lindenbach & Rice. Journal of Virology. 1997;71:9608-9617, PMID: 9371625)<br>Vero E6 cells (African green monkey kidney cells; ATCC® CRL-1586™)<br>C6/36 cells (mosquito cells; ATCC® CRL-1660™)<br>Huh-7 cells (Human hepatoma cells; provided by Prof. Ralf Bartenschlager, Heidelberg University, Germany; PMID: 6286115)<br>A549 cells (human lung carcinoma cells; ATCC CCL-185)
Authentication	No authentication of the cell lines was performed.
Mycoplasma contamination	All cell lines tested negative for mycoplasma contamination.<br>Absence of mycoplasma contamination was controlled using a cell-based commercial assay (Invivogen PlasmoTest™ Mycoplasma contamination detection kit; Cat. No. rep-pt1).
Commonly misidentified lines (See ICLAC register)	None of the commonly misidentified cell lines were used.
